# Prediction of Binding Stability of Pu(IV) and PuO_2_(VI) by Nitrogen Tridentate Ligands in Aqueous Solution

**DOI:** 10.3390/ijms21082791

**Published:** 2020-04-17

**Authors:** Keunhong Jeong, Hye Jin Jeong, Seung Min Woo, Sungchul Bae

**Affiliations:** 1 Department of Chemistry, Nuclear & WMD Protection Research Center, Korea Military Academy, Seoul 01805, Korea; hyejinj@swu.ac.kr; 2 Department of Nuclear and Energy Engineering, Jeju National University, Jeju 63243, Korea; woosm@jejunu.ac.kr; 3 Department of Architectural Engineering, Hanyang University, Seoul 04763, Korea; sbae@hanyang.ac.kr

**Keywords:** plutonium, extractant, tridentate, sensor, complexation

## Abstract

Plutonium has potential applications in energy production in well-controlled nuclear reactors. Since nuclear power plants have great merit as environmentally friendly energy sources with a recyclable system, a recycling system for extracting Pu from spent fuels using suitable extractants has been proposed. Pu leakage is a potential environmental hazard, hence the need for chemical sensor development. Both extractants and chemical sensors involve metal–ligand interactions and to develop efficient extractants and chemical sensors, structural information about Pu ligands must be obtained by quantum calculations. Herein, six representative nitrogen tridentate ligands were introduced, and their binding stabilities were evaluated. The tridentate L6, which contains tri-pyridine chelate with benzene connectors, showed the highest binding energies for Pu(IV) and PuO_2_(VI) in water. Analysis based on the quantum theory of atoms in molecular analysis, including natural population analysis and electron density studies, provided insight into the bonding characteristics for each structure. We propose that differences in ionic bonding characteristics account for the Pu-ligand stability differences. These results form a basis for designing novel extractants and organic Pu sensors.

## 1. Introduction

Plutonium is very useful for energy production in a carefully controlled nuclear reactor. Currently, approximately 14% of global electricity production is derived from nuclear reactors [1]. Nonetheless, a major drawback of this strategy is the generation of a large amount of radioactive waste, usually a mixture of U, Pu, and other actinides [2]. For example, about 2000 metric tons of spent nuclear fuel are annually generated in the United States [3]. Moreover, the Oklo natural nuclear reactor in Gabon went critical around 2 billion years ago. According to Gauthier-Lafaye’s paper, the experimental data proved retention of fission products and plutonium in Gabon [4].

The nuclear power industry faces challenges related to the irradiation of nuclear fuels by used fuel recycled by a pyroprocessing system or PUREX (Pu and U Extraction) system. Liquid waste from these systems contains highly toxic actinides, including Pu, which must be effectively extracted for reuse via a closed-loop recycling program, such that it does not contaminate the environment [5,6,7,8,9]. The efficiency of such programs depends on effective recycling of the spent nuclear fuel by separating the fissile Pu, which is formed by neutron capture reactions and is intended for subsequent use in a fast neutron reactor. Leakage of even small amounts of nuclear waste is undesirable, because the radioactive materials pose environmental problems owing to their long half-lives [10]. Therefore, it is imperative to develop efficient extractants and chemical sensors, such as fluorescence sensors (which fluoresce upon binding with the target metal ion) [11,12]. Therefore, investigating the binding and structures formed between toxic actinides and organic ligands is essential for understanding various modes of complexation.

Actinide partitioning using selectively binding organophosphorus ligands like tributyl phosphate (TBP), octyl-(phenyl)-*N*,*N*-diisobutylcarbamoylmethyl phosphine oxide (CMPO), trialkyl phosphine oxide (TRPO), and diisodecyl phosphoric acid (DIDPA), along with diamide extractants like *N,N*′-dimethyl-N,N′-dioctylhexylethoxy malonamide (DMDOHEMA) and *N*,*N*′-dimethyl-*N*,*N*′-dibutyltetradecyl malonamide (DMDBTDMA) [13,14,15,16,17,18,19,20], has been proposed. The extractants usually have a nitrogen (soft) donor in their ligand structure. Therefore, a systematic study on various kinds of nitrogen donor ligands with Pu should be informative for further study on new types of extractants.

While numerous experimental studies on the complexation of actinides using the appropriate extractants in a PUREX system have been reported, there have been hardly any theoretical studies that provide structural information on such complexes in aqueous solution [21]. Molecular-level investigations via reliable and extensive quantum calculations such as density functional theory (DFT) can provide valuable information for the design of efficient extractants for actinide separation in the environment, as well as new organic ligands for actinide detection. Pu mainly exists in the +4 and +6 oxidation states in the environment (oxygenated system), and Pu(VI) is easily oxidized to PuO_2_^2+^ in water [22].

**Figure 1:**
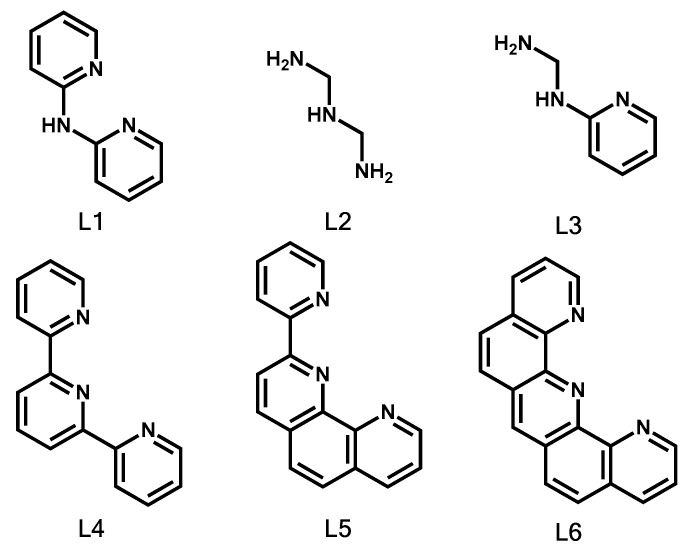
Structures of the six tridentate nitrogen ligands.

Therefore, in this study, attempts have been made to understand the contrasting selectivity for Pu in aqueous solution using a soft donor, nitrogen, thus revealing structural information related to Pu complexation. Tridentate nitrogen donor ligands are employed as binders and extractants for lanthanides and actinides. The most commonly used N-donor ligands and those basic structures are utilized for this study (Figure 1). This research aims to clarify the differences in the binding capabilities of six tridentate soft nitrogen donor ligands with Pu (IV) and PuO_2_ (VI) in water, and to investigate their bonding characteristics. 

## 2. Results and Discussion

### 2.1. Complexation and Bond Formation

**Figure 2:**
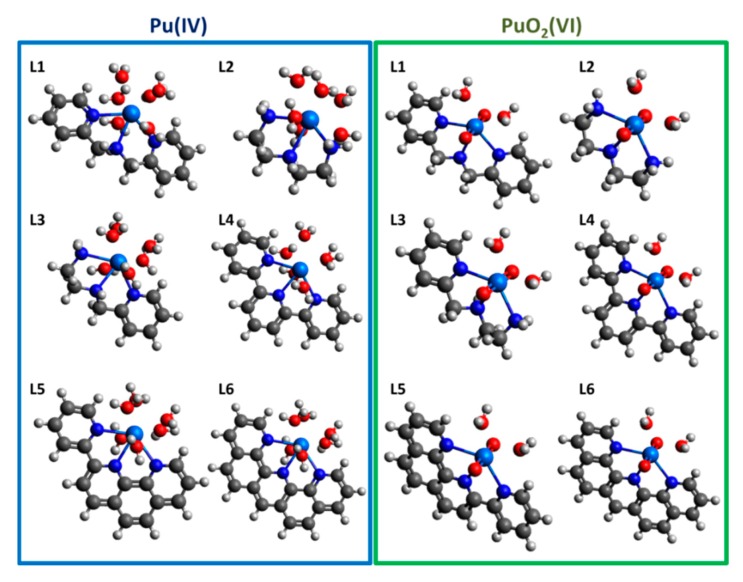
Optimized structures of Pu (IV) and PuO_2_(VI) with the suggested nitrogen tridentate ligands in aqueous solution (structural chemical formulas and coordinates of all structures are listed in SI).

Both Pu(IV) and PuO_2_(VI) form 1:1 complexes with tridentate ligands in a strongly solvating solvent like water to afford nonahydrates. The inner coordination sphere of the metal in the complexes comprises water molecules. For PuO_2_(VI), since the two oxygens occupy axial positions, the ligands interact with Pu in the equatorial plane, where the Pu can accommodate 4 more electron donors. Thus, the 1:1 complexes of Pu(VI) and Pu(IV) are filled with two and six water molecules, respectively [23]. Therefore, because of the lack of information about the target compound, we assessed the quality of the level of theory which was implemented and the same level of theory except the 6-31+G(d, p) for N, O, C, and H, respectively. Recently, the average Pu-N_amin_ and Pu-N_amido_ bond distances in Pu(IV)-triamidoamine(TREN) were measured to be 2.577 and 2.225 Å, respectively [24]. Quantum calculations on Pu(IV)-triamidoamine(TREN) without TIPS (Triisoproplysilane) were performed by using both of levels of theory. The average bond distances were found to be 2.606 (2.623) and 2.195 (2.199) Å, which is a slight overestimation (less than 1.5%) of the experimental data (structure and coordinates are presented in Supplementary Materials, Figure S2). Another study with reliable and extensive quantum calculations using a TPSSH functional with the Stuttgart small core ECP and def2-TZVP basis set on plutonium complexes has been carried out [25]. Therefore, the Pu(IV)-triamidoamine(TREN) structure was also optimized using the same level of theory, which also showed almost the same overestimation as the previous one (structure and coordinates are presented in Supplementary Materials, Figure S2). Therefore, here we performed the theoretical study at both levels of theory, both the computationally less expensive and the more expensive. With the same levels of theory, the optimized structures of each complex with water molecules are presented in Figure 2, and the average bond lengths are listed in Table 1. The Pu(IV)–N bonds are shorter than the corresponding PuO_2_ (VI)–N bonds, because the interaction between Pu(IV) and N is stronger than that in the other complexes in both calculations. This difference could stem from the fact that Pu(VI) forms a PuO_2_^2+^ complex with weak positive charges on the central metal ion, which decrease the strength of the ionic bonding. The Pu–O (oxygen in the coordinating water molecule) bond length shows a different trend, because the oxidized structure of Pu(VI) has less steric hindrance between ligands, though the atomic charges of Pu(VI) and O are smaller than others. This hypothesis is well supported by the NPA analysis discussed in the supporting information.

### 2.2. Comparison of Stability

The reactions below denote the complex formation reaction for each Pu oxidation state.

Lx + Pu(IV) + 6H_2_O→PuLx(H_2_O)_6_

Lx + PuO_2_^2+^(VI) + 2H_2_O→PuO_2_Lx(H_2_O)_6_

(x = 1, 2, 3, 4, 5, 6)

To quantitatively estimate the strength of stability for the complexes in aqueous solution, all possible spin multiplicities are considered, because most of the unpaired electrons reside in the 5f orbitals and lead to different multiplicities depending on the ligand binding. The lowest energy was observed for the triplet state in the case of the Pu(IV) structures, and for the quintet state in the case of the PuO_2_(VI) structures (see Table S4 in Supplementary Materials for details of the calculated energy for each spin multiplicity). Since the reactants with Pu (PuO_2_) and the water molecules are the same in each reaction, the energy difference from the lowest energy was used for calculating the stabilization energy for each functional: E_diff_ = E [PuL6(H_2_O)_6_] − (E [PuLx(H_2_O)_6_] − E [Lx]) and E_diff_’ = E [PuL6(H_2_O)_6_] − (E [PuO_2_(H_2_O)_2_] − E [Lx]), (x = 1, 2, 3, 4, 5, 6). According to the analysis, L6 formed the most stable complex with both Pu(IV) and PuO_2_^2+^(VI) species in both levels of theory (Table 2).

**Figure 3:**
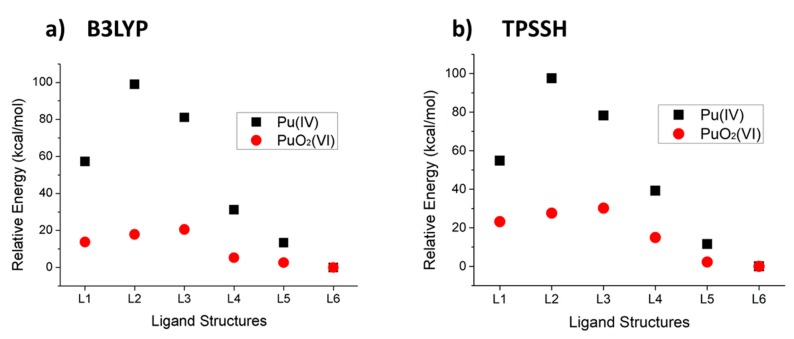
Relative energy of each structure compared with the most stable complex (Pu–L6) using different functionals: (**a**) B3LYP, (**b**) TPSSH.

After applying two different functionals and basis sets, of all the optimized structures the L6 complex was the most stable, and its stability trend for the series of studied ligands is very similar to all except the L2 and L3 cases, indicating that complexation with the pyridine nitrogen on rigid scaffold leads to high stability and Pu complexation is strongly dependent on the ligand structures (Figure 3). It is possible that the ligand complexation properties are almost the same in both Pu(IV) and Pu(VI), based on QTAIM analysis of the bonding properties. L6 may be a better extractant for Pu(IV) than for PuO_2_(VI), as it showed a much bigger energy difference between Pu(IV) and L6 in aqueous solution. In addition, L6 would selectively extract Pu(IV) ions in a solution containing various other metal ions and ligand-like materials. Its stability of complexation shows its tendency to increase stability on more rigid structures, which implies a nitrogen-doped graphene structure could be a key structure toward extracting plutonium in water. Nitrogen doping technology has been developed in various fields [26], and it may be a good start for application of graphene-based materials in nuclear chemistry in the future.

### 2.3. Electronic Structure Analysis

Wiberg bond indices are a direct indicator of covalency [27]. Generally, Pu binding with a primary amine results in a larger Wiberg index, indicating more strongly covalent characteristics, which is the same trend as those of the other indicators described below. One can thus conclude that stronger ionic bonding is related to the stability of the complex formed with a secondary amine such as pyridine. The NPA charge values (Table S1) show that all the Pu atoms act as electron receptors, which carry a charge of less than 4 + (Pu(IV)) or 2 + (PuO_2_(VI)). Interestingly, the change of charges on Pu(IV) after forming complexes, from 4 to 1.43~1.54, is greater than the change of charge on PuO_2_(VI) cases, from 2 to 1.26~1.30. This is closely related to the bonding properties, which can be attributed to more covalent bonding forms in the Pu(IV) structures. This is strongly supported by the following topology analysis. Furthermore, the populations of the 7s, 7p, 5f, and 6d atomic orbitals of both structures differ from the canonical numbers (most electrons reside on 5f orbital), indicating that electron transfer from the ligand to Pu makes a major contribution to the ionic bond (see Supplementary Materials, Table S3). 

Topology analysis was carried out to investigate each bond more closely. A BCP (3, −1) existed between two binding atoms (see Figure S1 and Table S2 in Supplementary Materials for BCPs). Generally, the electron density at the BCP is used as a standard for evaluating the bonding characteristics. A larger ρ(r) and smaller ∇^2^ρ(r) indicate a more covalent bond, while a smaller ρ(r) and larger ∇^2^ρ(r) indicate an ionic bond. This information clarified that Pu(IV)–N bonds had a stronger covalent nature than PuO_2_ (VI)–N bonds. In addition, the −V(r)/G(r) ratios (G(r): local kinetic energy density, V(r): local potential energy) are shown in Table 3. A − V(r)/G(r) ratio of less than 1 indicates a typical ionic bond, while a ratio greater than 1 represents a covalent bond. Accordingly, Pu(IV) showed stronger covalent bonding characteristics than PuO_2_(VI). There was no significant difference in the Pu–O bond between the two structures. Even though the two structures did not show exactly the same trend of ligand stability, we may conclude that stronger ionic bonding results in better stability of the complexes. Moreover, these studied bonding properties can be harnessed to extract specific oxidation states of plutonium in water and, in future research, this kind of investigation on bonding properties can be utilized in the development of new types of extractants by comparing different functional groups in ligands.

## 3. Computational Details

The computational level of B3LYP functional that is mainly considered in our study has recently been demonstrated to be reliable for actinide complexes [28,29,30]. The B3LYP functional and TPSSH were also utilized for geometry optimization in the gas phase, with tight energy and geometry convergence criteria, and without symmetry constraints. The ECP60MWB relativistic effective core potential and its associated basis set, developed by the Stuttgart–Cologne group, was chosen for describing plutonium, while 6-31G(d) for B3LYP functional and def2-TZVP for TPSSH functional were used for N, O, C, and H, respectively. These basis sets have been successfully utilized for describing actinide-organic complexes including plutonium in recent reports [31,32,33,34]. Spin-orbit effects were not considered, because the quasi-relativistic effective core potential takes into account the spin-orbit coupling effect and produces reliable electronic structures for studies on actinide complexes. To compare all structures under the same conditions, the initial ligand structures were required to be non-protonated. After optimization by B3LYP functional, the local minimum structure was confirmed by checking all positive frequencies. The theoretical calculations were performed using the Gaussian 09 software package [35]. Bader’s atoms-in-molecule (AIM) model, natural population analysis (NPA) of charge, and topological analysis of electron density can provide information about chemical bonds through bond critical point (BCP) positions. These methods were adopted here to understand the nature of bonding by estimating the bond strengths and electron density in the bonds. Analysis based on the quantum theory of atoms in molecules (QTAIM) was carried out using the Multiwfn program [36].

## 4. Conclusions

In this work, representative nitrogen tridentate ligands were introduced and their binding properties were investigated by using two different levels of theory to obtain information that will aid the design of plutonium extractants and metal ion sensors in aqueous solution. Pu(IV) and PuO_2_^2+^(VI) with six tridentate ligands in solution were simulated. Interestingly, L6, which is a tri-pyridine chelate with benzene connectors, showed the highest binding energy for both Pu(IV) and PuO_2_(VI) ions in water. QTAIM analysis and NPA studies revealed the nature of the Pu–N and Pu–O bonds, and showed that highly ionic bonds account for the enhanced bonding strength and, consequently, the stability of these complexes. L6-like structures might be promising candidates for strong extractants and sensors in water, and L6-based structures may be used as platforms for designing novel extractants and Pu sensors. More importantly, since studies on radioactive actinides have safety limitations, alternative and safe theoretical studies can provide more insight on the nature of actinide chemistry before doing experiments, which will open up new opportunities to discover new chelating agents and sensors in the future.

## Figures and Tables

**Table 1:** Average bond length (Å) of Pu–tridentate structures. Each number shows the distance between Pu and N or O in the optimized structure.

Ligand	B3LYP				TPSSH			
	Pu(IV)		PuO_2_(VI)		Pu(IV)		PuO_2_(VI)	
	Pu–N	Pu–O	Pu–N	Pu–O	Pu–N	Pu–O	Pu–N	Pu–O
L1	2.488	2.509	2.536	2.482	2.501	2.529	2.522	2.473
L2	2.544	2.504	2.558	2.476	2.561	2.509	2.551	2.467
L3	2.509	2.513	2.5473	2.483	2.574	2.530	2.536	2.478
L4	2.457	2.511	2.516	2.498	2.560	2.562	2.502	2.489
L5	2.498	2.529	2.517	2.512	2.570	2.571	2.499	2.533
L6	2.518	2.535	2.524	2.508	2.600	2.581	2.506	2.535

**Table 2:** Calculated energy difference compared with L6 ligand energy (kcal/mol) for each structure using both functionals.

Ligand	B3LYP		TPSSH	
	Pu(IV) (E_diff_)	PuO_2_(VI) (E_diff_’)	Pu(IV) (E_diff_)	PuO_2_(VI) (E_diff_’)
L1	57.251	13.724	54.775	23.182
L2	98.955	17.835	97.511	27.587
L3	81.101	20.506	78.194	30.178
L4	31.228	5.288	39.219	14.959
L5	13.362	2.564	11.546	2.240
L6	0	0	0	0

**Table 3:** Wiberg indices and −V(r)/G(r) ratios used for analyzing bond characteristics.

Ligand	Pu(IV)				PuO_2_(VI)			
	Wiberg Index		−V(r)/G(r)		Wiberg Index		−V(r)/G(r)	
	Pu–N	Pu–O	Pu–N	Pu–O	Pu–N	Pu–O	Pu–N	Pu–O
L1	0.414	0.278	1.121	0.919	0.369	0.317	0.929	0.894
L2	0.794	0.727	1.170	0.985	0.716	0.608	1.053	0.958
L3	0.774	0.574	1.048	0.950	0.355	0.317	0.928	1.275
L4	0.420	0.275	1.143	0.929	0.349	0.313	0.921	0.898
L5	0.734	0.559	1.003	0.947	0.348	0.307	0.923	0.897
L6	0.389	0.279	1.049	0.917	0.345	0.311	0.921	1.650

## Supplementary Materials

The following are available online at https://www.mdpi.com/1422-0067/21/8/2791/s1.
