# Supplementary material for: Prediction of Binding Stability of Pu(IV) and PuO2(VI) by Nitrogen Tridentate Ligands in Aqueous Solution
Source: Int J Mol Sci. 2020 Apr 17;21(8):2791. doi: 10.3390/ijms21082791 (PMC7216098; doi:10.3390/ijms21082791)
Supplement: Supplementary file 1 [file ijms-21-02791-s001.pdf]

Supporting Information

Prediction of binding stability of Pu(IV) and PuO_2(VI) by nitrogen tridentate ligands in aqueous solution

Keunhong Jeong [1,*], Hye Jin Jeong [1], Seung Min Woo [2] and Sungchul Bae [3]

1. Department of Chemistry, Nuclear & WMD Protection Research Center, Korea Military Academy, Seoul 01805, Korea; doas1mind@kma.ac.kr
2. Department of Nuclear and Energy Engineering, Jeju National University, Jeju 63243, Korea; woosm@jejunu.ac.kr
3. Department of Architectural Engineering, Hanyang University, Seoul 04763, Korea; sbae@hanyang.ac.kr

* Correspondence: doas1mind@kma.ac.kr or doas1mind@berkeley.edu; Tel.: +82+2-2197-2823

**Pu(IV)-L1**	**PuO_2(VI)-L1**
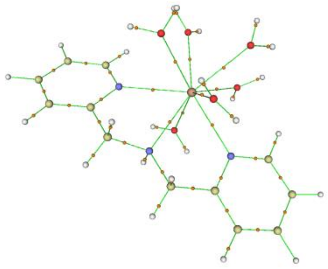	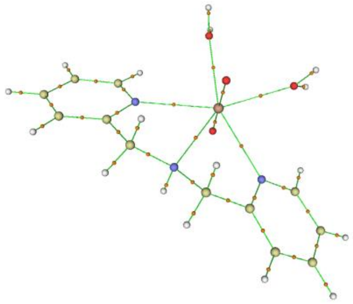
**Pu(IV)-L2**	**PuO_2(VI)-L2**
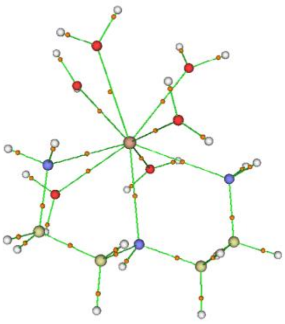	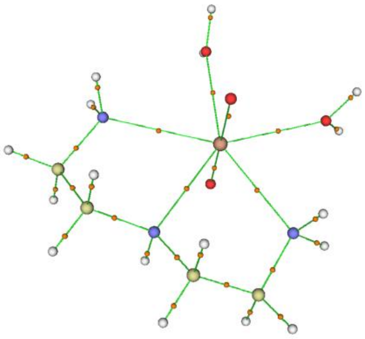
**Pu(IV)-L3**	**PuO_2(VI)-L3**
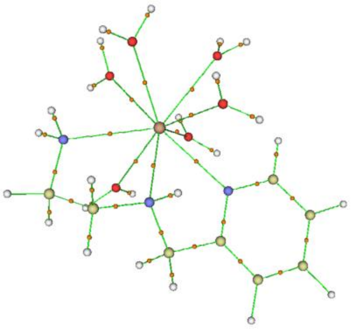	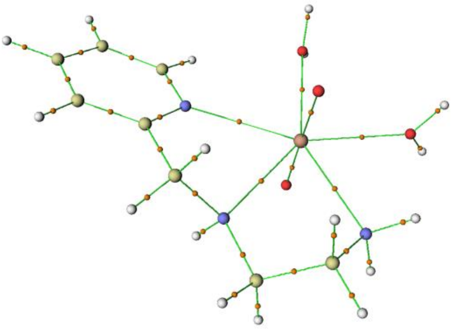
**Pu(IV)-L4**	**PuO_2(VI)-L4**

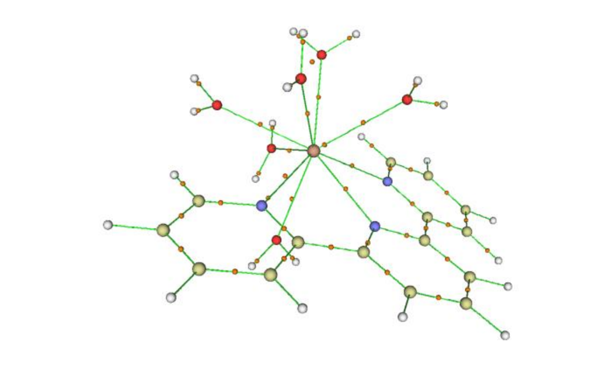	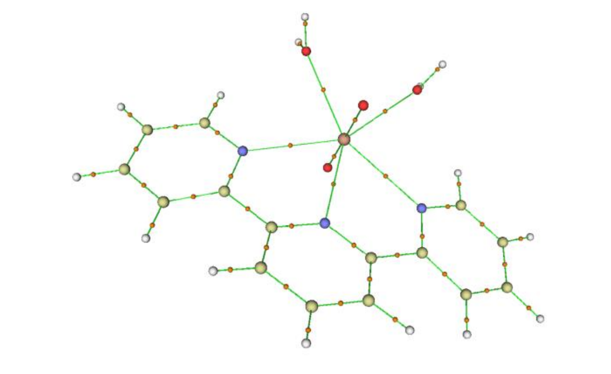
**Pu(IV)-L5**	**PuO_2(VI)-L5**
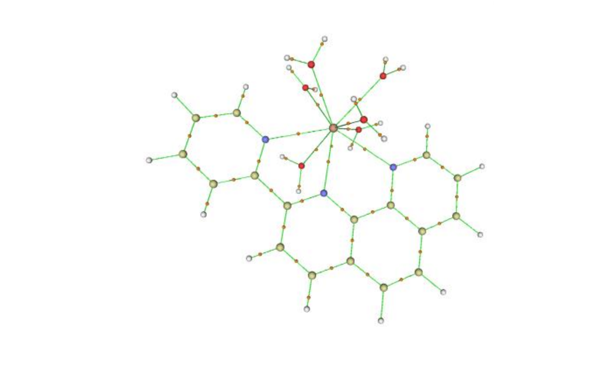	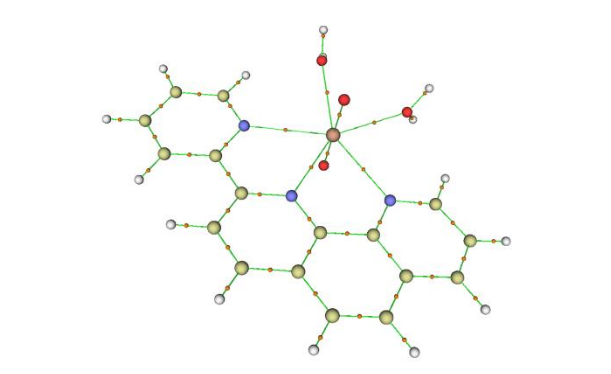
**Pu(IV)-L6**	**PuO_2(VI)-L6**
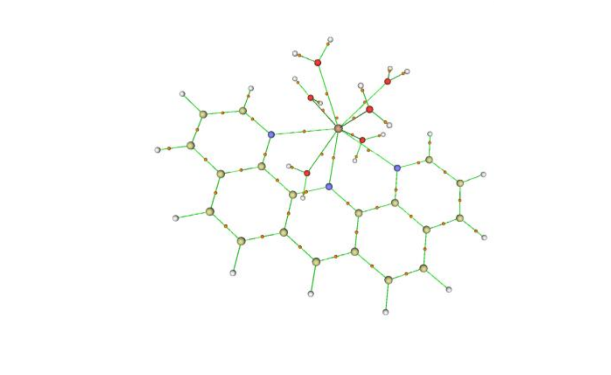	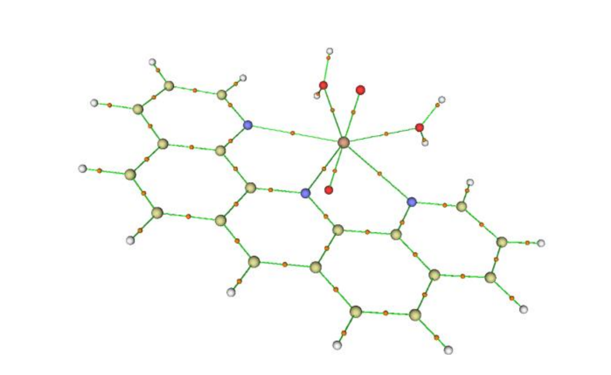

Figure S1. BCP (3-1) for studied structures

Table S1. NPA for Pu, N, and O in the optimized structures

	Pu(IV)			PuO_2(VI)		
	Pu	N	O	Pu	N	O
**L1**	1.43244	−0.57591	−0.88846	1.26875	−0.53374	−0.86669
**L2**	1.54146	−0.80628	−0.90328	1.29779	−0.78603	−0.87226
**L3**	1.44838	−0.68922	−0.89227	1.29234	−0.66218	−0.87212
**L4**	1.45657	−0.52344	−0.8902	1.27732	−0.45847	−0.87379
**L5**	1.43804	−0.51917	−0.8962	1.29494	−0.4564	−0.87795
**L6**	1.43709	−0.51773	−0.90002	1.30037	−0.45409	−0.87741

Table S2. Electron density and Laplacian of Pu-N and Pu-O bonds

			L1	L2	L3	L4	L5	L6
**Pu(IV)**	Pu-N	ϱ(r)	0.044866	0.059181	0.057592	0.049318	0.053902	0.041486
		∇^2ϱ(r)	0.204654	0.121572	0.174666	0.232226	0.200623	0.203479
	Pu-O	ϱ(r)	0.030118	0.045003	0.040705	0.032428	0.038992	0.028798
		∇^2ϱ(r)	0.181641	0.176151	0.180182	0.181165	0.172312	0.171130
**PuO_2(VI)**	Pu-N	ϱ(r)	0.032760	0.050552	0.031802	0.033000	0.033295	0.032787
		∇^2ϱ(r)	0.239711	0.155336	0.226624	0.249527	0.248580	0.244395
	Pu-O	ϱ(r)	0.029744	0.042591	0.196708	0.028222	0.027141	0.364151
		∇^2ϱ(r)	0.226968	0.210235	0.492794	0.227317	0.217938	0.772873

Table S3. Pu electron population in outer orbitals

	Pu(IV)	PuO_2(VI)
**L1**	7S(0.23)5f(4.91)6d(0.94)	7S(0.21)5f(5.08)6d(1.31)7p(0.34)
**L2**	7S(0.25)5f(4.81)6d(0.92)7p(0.27)	7S(0.21)5f(5.06)6d(1.31)7p(0.34)
**L3**	7S(0.24)5f(4.90)6d(0.92)	7S(0.21)5f(5.06)6d(1.31)7p(0.34)
**L4**	7S(0.24)5f(4.89)6d(0.94)	7S(0.21)5f(5.06)6d(1.33)7p(0.34)
**L5**	7S(0.24)5f(4.97)6d(0.88)	7S(0.22)5f(5.05)6d(1.32)7p(0.34)
**L6**	7S(0.24)5f(5.01)6d(0.86)	7S(0.22)5f(5.04)6d(1.32)7p(0.34)

Table S4. Calculated energy for each multiplicity for finding the most stable spin state

**Pu(IV)**						
	**L1**	**L2**	**L3**	**L4**	**L5**	**L6**
**Singlet**	−1640.829268	-1335.833973	-1488.334076	-1753.997541	-1830.270924	-1906.531030
**Triplet**	-1640.898466	-1335.904126	-1488.402521	-1754.074186	-1830.348461	-1906.617782
**Quintet**	-1640.944443	-1335.951003	-1488.448092	-1754.124155	-1830.399432	-1906.664823
**Septet**	-1640.920693	-1335.890199	-1488.416666	-1754.114858	-1830.395591	-1906.663505
**Nonet**	-1640.773216	-1335.686976	-1488.265247	-1753.987291	-1830.288712	-1906.576303
**PuO_2(VI)**						
	**L1**		**L1**		**L1**	
**Singlet**	-1486.509157	Singlet	-1486.509157	Singlet	-1486.509157	Singlet
**Triplet**	-1486.547165	Triplet	-1486.547165	Triplet	-1486.547165	Triplet
**Quintet**	-1486.505815	Quintet	-1486.505815	Quintet	-1486.505815	Quintet
**Septet**	-1486.382738	Septet	-1486.382738	Septet	-1486.382738	Septet
**Nonet**	-1486.220429	Nonet	-1486.220429	Nonet	-1486.220429	Nonet

Structure and coordinates of optimized Pu-TREN structure

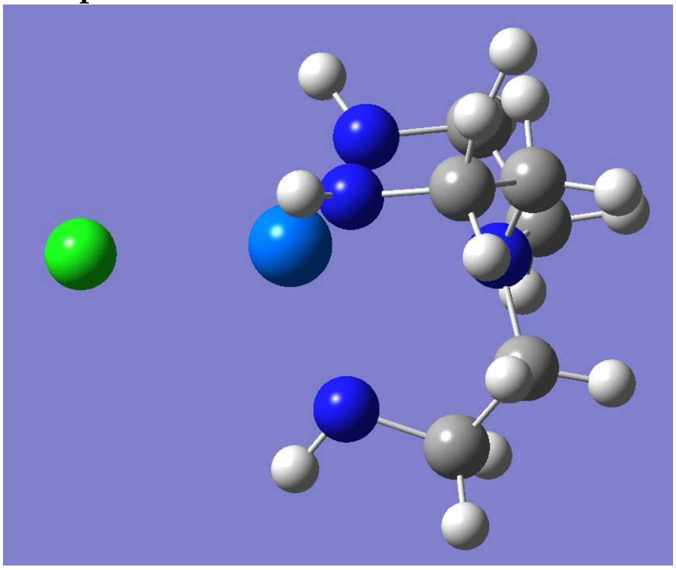

Figure S2. Pu-TREN structure

<B3LYP/6-31G(d)>

Pu	-0.63505	0.052603	-0.03122
Cl	-3.2565	-0.06189	0.013819
N	1.96701	-0.07885	0.040653
N	0.312251	1.402665	-1.48653
N	0.122188	0.656998	1.925581
N	0.06461	-1.9985	-0.42611
C	2.465423	0.316851	-1.29851
C	1.720902	1.551415	-1.81655
C	2.40005	0.867322	1.097587
C	1.512789	0.76235	2.343233
C	2.307367	-1.48457	0.366683
C	1.443377	-2.4677	-0.43351
H	2.2666	-0.51791	-1.97524
H	3.555315	0.483311	-1.28124
H	2.149669	2.470753	-1.38105
H	1.898908	1.623606	-2.9022
H	2.299449	1.874565	0.686513
H	3.461143	0.714006	1.355211
H	1.816928	-0.10186	2.958574
H	1.699853	1.653601	2.963989
H	2.097592	-1.63014	1.429643
H	3.381472	-1.67695	0.20713
H	1.837247	-2.57626	-1.45906
H	1.550667	-3.45973	0.03376
H	-0.50766	0.688685	2.730602
H	-0.23642	2.159371	-1.90422
H	-0.57712	-2.74773	-0.69109

<B3LYP/6-31++G(d,p)>

Pu	-0.63424500	0.04921100	-0.02813800
Cl	-3.25510800	-0.06462900	-0.01366600

N	1.96544700	-0.07984700	0.04201500
N	0.31130800	1.41407100	-1.47537000
N	0.12401600	0.65321700	1.93237800
N	0.06387500	-2.00526700	-0.43095300
C	2.46103900	0.31372800	-1.30009500
C	1.72158300	1.55339400	-1.81412100
C	2.40112100	0.86965700	1.09639700
C	1.51832400	0.76538000	2.34538400
C	2.30532100	-1.48638200	0.37041000
C	1.44688400	-2.47040300	-0.43481000
H	2.25627100	-0.51927500	-1.97690400
H	3.55172100	0.47477100	-1.28552000
H	2.15609100	2.47031700	-1.38120300
H	1.89055800	1.62323400	-2.90053100
H	2.29874200	1.87620300	0.68476800
H	3.46299000	0.71615700	1.35031300
H	1.82451600	-0.09606000	2.96213900
H	1.70152000	1.65946600	2.96186200
H	2.09059700	-1.63265600	1.43199800
H	3.38031900	-1.67687400	0.21481800
H	1.84062500	-2.57431600	-1.46008000
H	1.55342000	-3.46248700	0.03049900
H	-0.50424100	0.69475700	2.73643600
H	-0.23826400	2.16532600	-1.89767000
H	-0.57637500	-2.75469700	-0.69390800

<TPSSH/def2-TZVP>

Pu	-0.62282800	0.05478400	-0.03629800
Cl	-3.22023100	-0.04683700	0.03688200
N	1.93876400	-0.07986700	0.03852700
N	0.34085900	1.43598900	-1.44190800
N	0.13489000	0.58953700	1.93563700
N	0.09682600	-1.97177100	-0.47196900
C	2.43801100	0.27818200	-1.30662600
C	1.74063800	1.53582000	-1.81333600
C	2.36746500	0.89417600	1.06566300
C	1.52336900	0.75831500	2.32917200
C	2.28504500	-1.47056100	0.40080400
C	1.46548400	-2.45723300	-0.42609900
H	2.18570900	-0.55267300	-1.96717700
H	3.53186900	0.39546100	-1.30446600
H	2.21181300	2.43785200	-1.39316200
H	1.88668200	1.59618100	-2.90140300
H	2.20324000	1.88671100	0.64394700
H	3.43919100	0.78804200	1.29014500
H	1.87410400	-0.08760300	2.93972000
H	1.67810400	1.65892200	2.94013300
H	2.02203400	-1.59989500	1.45187400
H	3.36490600	-1.65003400	0.29036700
H	1.89706900	-2.57201300	-1.43267400
H	1.54093800	-3.44436300	0.05037300
H	-0.47845100	0.58319600	2.75053400
H	-0.18838500	2.22234100	-1.82218700
H	-0.53906200	-2.70666100	-0.78002700

Structural chemical formulas and its coordinates of optimized structures (B3LYP/6-31G(d))

Pu(IV)-L1

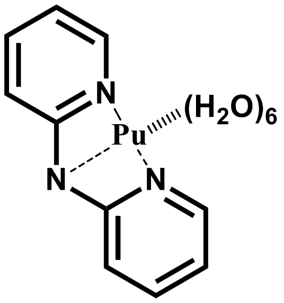

Pu	0.05649900	0.10531800	0.14569100
O	-1.09985100	0.60241200	-2.04273200
H	-2.05701600	0.50171000	-2.21586000
O	-0.49405100	-0.79060300	2.41308700
H	-1.23403500	-1.38864000	2.63567900
O	1.91942900	-1.23285400	1.13055400
H	1.86346800	-1.77896800	1.93864200
O	1.39383700	1.43092500	1.78522200
H	2.31056900	1.24476800	2.06946000
O	0.24523600	2.52254300	-0.59800500
H	0.84679100	3.21195100	-0.25454500
H	-0.10926400	-0.50212200	3.26465100
H	1.09067500	2.19097900	2.32003000
H	2.82776900	-1.33814000	0.78731500
H	-0.18643000	2.90979400	-1.38410900
H	-0.69284900	0.83261600	-2.90180000
N	0.02434700	-2.01509900	-1.13429000
N	-2.14446200	-1.09842600	0.09911000
N	1.90250300	-0.12155000	-1.49367900
C	-3.35473800	-0.50272500	0.30634700
C	-4.54923400	-1.20948300	0.34979700
C	-4.52097900	-2.59858900	0.17204800
C	-3.29054500	-3.22122900	-0.07063400
C	-2.13102200	-2.45379700	-0.10793500
C	-0.78297700	-3.05209000	-0.42529600
C	2.70001100	0.93766000	-1.82553200
C	3.76448400	0.83711300	-2.70989300
C	4.03418100	-0.40114600	-3.30333400
C	3.22588700	-1.49591300	-2.97417200
C	2.18341600	-1.33633400	-2.06658400
C	1.34296800	-2.50747200	-1.62653200
H	-5.48309200	-0.68122800	0.51922800
H	-5.43801300	-3.18199700	0.20112300
H	-3.24018500	-4.29242600	-0.24646200
H	-0.88779700	-3.94809700	-1.04972800
H	-0.23869000	-3.36098400	0.47659500
H	4.36331800	1.71511500	-2.93393700
H	4.85347400	-0.51358100	-4.00937500
H	3.40854100	-2.46946600	-3.42159900
H	1.19624000	-3.21981300	-2.44747000
H	1.82900900	-3.05841300	-0.81397600
H	2.46091600	1.88999400	-1.37030200
H	-3.35091600	0.57233100	0.44375700
H	-0.51302100	-1.73040300	-1.95912100
O	-1.41513900	1.71800900	1.36846700

H	-1.94610800	1.57554300	2.17649000
H	-1.61257200	2.62375700	1.05890800

Pu(IV)-L2

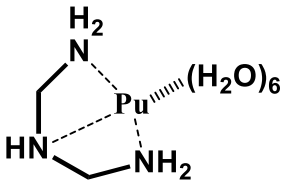

Pu	-0.12025400	-0.26940300	0.18450900
O	-1.99772900	0.08214800	-1.50235300
H	-2.76685500	0.63338000	-1.24999300
O	-0.85806600	-0.12777300	2.65682000
H	-1.64712700	-0.45098600	3.13977500
O	0.98056800	-1.88242100	1.67124400
H	0.74976200	-1.88125100	2.62342600
O	1.32418800	1.35256700	1.37561100
H	2.20302300	1.22072500	1.79083700
O	0.37768000	1.40405500	-1.56053000
H	1.12137100	2.03245900	-1.67296300
H	-0.38270100	0.43025700	3.30632900
H	1.11429700	2.29989100	1.51354300
H	1.62131300	-2.61468800	1.55656200
H	-0.23604000	1.58443000	-2.30147800
H	-2.24861500	-0.30937500	-2.36437100
N	0.10974800	-2.27452100	-1.37529400
N	-1.92791200	-1.99605600	0.59072400
N	2.27147400	-0.59011400	-0.63788500
C	-2.09459700	-3.01522300	-0.51575900
C	-0.72318800	-3.46928400	-0.97334900
C	2.37353500	-1.42209600	-1.89479800
C	1.53024100	-2.66753200	-1.71201800
H	-0.80604600	-4.16540800	-1.81777600
H	-0.19093100	-3.99400000	-0.17389200
H	1.53749600	-3.28224900	-2.62113700
H	1.90897800	-3.29901400	-0.90098100
H	-0.28903100	-1.92388300	-2.25584300
H	2.02381400	-0.81420500	-2.73521500
H	3.41780500	-1.68637600	-2.09677700
H	2.86126400	-1.03185700	0.07661500
H	2.74890200	0.29993300	-0.81557100
H	-1.70140700	-2.51668500	1.44606600
H	-2.85184400	-1.58552600	0.77489000
H	-2.68230700	-3.86728900	-0.15434900
H	-2.66713500	-2.54964900	-1.32151000
O	-1.47539700	1.76316200	0.68395300
H	-1.97477300	1.91952200	1.51166200
H	-1.53656100	2.59808200	0.17429500

Pu(IV)-L3

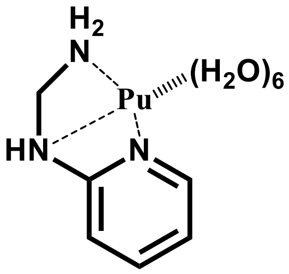

Pu	0.09945400	-0.35755200	0.21655100
O	-1.16836600	-0.15425400	-1.86096500
H	-1.91508100	-0.60658500	-2.30166700
O	-0.52905600	-0.51226000	2.67415800
H	-1.11917500	-1.08285100	3.20746500
O	1.89485900	-1.42718600	1.62272800
H	1.76845400	-1.76357200	2.53250400
O	1.33210200	1.32655200	1.58887900
H	2.11603200	1.13268100	2.14082600
O	0.47474400	1.79456400	-1.19040900
H	1.25922800	1.98249800	-1.74279200
H	-0.17614000	0.14481500	3.30730100
H	1.25843100	2.30196500	1.56055500
H	2.81885100	-1.64013400	1.38260700
H	0.03843200	2.66064200	-1.06348700
H	-0.88246200	0.56684600	-2.45868400
O	-1.60939200	1.36352200	0.73413300
H	-1.89914000	1.62433900	1.63218000
H	-2.26800600	1.75707100	0.12566200
N	0.31464800	-2.77086600	-0.54286700
N	1.97724600	-0.77777700	-1.24491800
N	-2.09436100	-1.68804400	0.38554300
C	3.05307300	0.06815600	-1.28555100
C	4.08893600	-0.08343700	-2.19351200
C	4.03807400	-1.15278700	-3.10515300
C	2.96077900	-2.04406100	-3.04771000
C	1.95240900	-1.84488500	-2.10892800
C	0.79304500	-2.79674800	-1.96130500
C	-2.15976800	-2.93140800	-0.46664000
C	-0.85902600	-3.68694200	-0.28212700
H	4.92116800	0.61530100	-2.18993600
H	4.83644400	-1.29992500	-3.82942100
H	2.91838500	-2.90028600	-3.71618300
H	1.09268400	-3.81596800	-2.23589400
H	-0.03299900	-2.52172800	-2.62666500
H	-0.76774200	-4.05716300	0.74469100
H	-0.81108000	-4.56159000	-0.94113400
H	3.06931300	0.87831600	-0.56115800
H	1.09030100	-3.12564200	0.02367400
H	-3.01515200	-3.55374900	-0.18148100
H	-2.31836200	-2.63920200	-1.50880600
H	-2.91431000	-1.10576700	0.18382700
H	-2.26179600	-1.99048500	1.35116100

Pu(IV)-L4

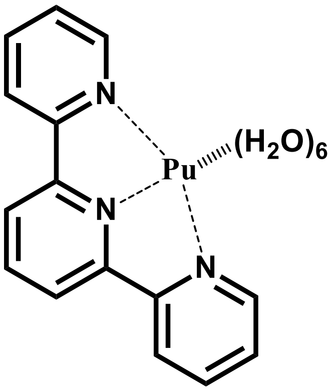

Pu	0.19784600	-0.12238500	0.22943500
O	-0.75938300	0.07472900	-2.06497700
H	-1.62220400	-0.32157400	-2.29576400
O	-0.48982800	-0.87432900	2.51987200
H	-1.16083000	-1.54846900	2.74039100
O	2.07464600	-1.16938000	1.54262700
H	1.91599400	-1.78184600	2.28624500
O	1.28216300	1.41524900	1.86584700
H	2.13258800	1.21474200	2.30313200
O	0.21963100	2.20899000	-0.78803400
H	0.67344200	3.03640400	-0.53671700
H	-0.20597400	-0.48004900	3.36786900
H	0.96327100	2.25734400	2.24503400
H	2.99324300	-1.32619400	1.25356100
H	-0.19340900	2.37743100	-1.65691700
H	-0.24414300	0.08671500	-2.89566400
N	0.56160100	-2.29500900	-0.78293900
N	2.22778000	-0.20751200	-1.14170100
N	-1.85258900	-1.53729300	0.09877700
C	3.02137300	0.87580200	-1.33259700
C	4.21156900	0.83297200	-2.05434500
C	4.60890500	-0.38370100	-2.61559700
C	3.81120400	-1.51304400	-2.41187900
C	2.63146600	-1.41499300	-1.66540100
C	1.75433800	-2.56780700	-1.39598500
C	-3.10664200	-1.08618900	0.36157600
C	-4.21839700	-1.91899500	0.44793700
C	-4.03857800	-3.29299700	0.27199500
C	-2.75923500	-3.77113100	-0.02594500
C	-1.68564900	-2.88171800	-0.12867800
C	-0.34292100	-3.29596000	-0.58623000
H	4.79889700	1.73738700	-2.18385200
H	5.52310900	-0.45632500	-3.19931900
H	4.11883100	-2.45923200	-2.84138500
H	-5.19754000	-1.49566400	0.65180400
H	-4.87726600	-3.98041600	0.34601200
H	-2.62275200	-4.83241700	-0.19662600
H	-3.21620000	-0.01804100	0.50261600
H	2.68080900	1.81164600	-0.90755400
C	2.10222500	-3.88015200	-1.74419300
C	-0.02440900	-4.62380500	-0.90192200

C	1.21576500	-4.91913900	-1.46986000
H	3.05431500	-4.10215000	-2.21112600
H	1.47645600	-5.94495600	-1.71687500
H	-0.73495600	-5.42420100	-0.73389500
O	-1.49612400	1.47787800	1.15758100
H	-1.75941000	2.30480600	0.70884800
H	-2.02180000	1.42376800	1.97919700

Pu(IV)-L5

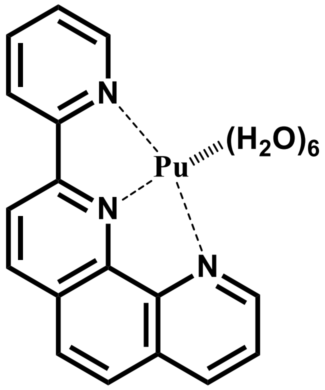

Pu	0.11758600	-0.10977800	0.21248500
O	-1.18869600	-0.03882600	-1.97654400
H	-2.11812500	0.25934800	-2.01480000
O	-0.69476900	-0.06333700	2.61783900
H	-1.28862000	-0.67100400	3.09802000
O	1.57684000	-1.31602000	1.77648600
H	1.30335000	-1.57845800	2.67596300
O	1.34304900	1.66791400	1.57651000
H	2.08052300	1.47798700	2.18842300
O	0.41852400	1.96965800	-1.26496000
H	1.09088200	2.67660400	-1.26894400
H	-0.47764000	0.65467600	3.24227200
H	1.15443200	2.62144200	1.67648200
H	2.40223000	-1.79718000	1.57917200
H	0.12795000	1.86949900	-2.19182800
H	-1.08410400	-0.65925300	-2.72221600
N	0.54729000	-2.25362800	-0.92495300
N	2.42322700	-0.28695000	-0.79446100
N	-1.73496100	-1.78677300	0.43349800
C	3.37729000	0.66915500	-0.71173700
C	4.59970100	0.61124700	-1.38774800
C	4.87349000	-0.49035300	-2.18683200
C	3.91242000	-1.52836000	-2.28679100
C	2.69050100	-1.37945800	-1.57094700
C	1.70640800	-2.42705800	-1.62275800
C	-2.89207500	-1.50285100	1.07125900
C	-3.94285400	-2.41008200	1.21539800
C	-3.79214800	-3.68724200	0.67721200
C	-2.61149700	-3.99032700	-0.00723900
C	-1.60080100	-3.02726000	-0.13194000
C	-0.36304300	-3.27113400	-0.89449900
H	5.31705000	1.41856900	-1.27591700
H	5.81692100	-0.56766000	-2.72238700
H	-4.84708100	-2.11614200	1.74015700

H	-4.57707500	-4.43247900	0.77470500
H	-2.50038400	-4.97584800	-0.44243900
H	-2.98754300	-0.50559900	1.48750600
H	3.15438300	1.51794200	-0.07481800
C	1.98962600	-3.61068200	-2.36376600
C	-0.13665100	-4.47589600	-1.59325400
C	1.01916500	-4.64446700	-2.33025200
H	1.19007500	-5.56877000	-2.87694000
H	-0.86451000	-5.27661300	-1.56930000
O	-1.64180400	1.66028400	0.41211300
H	-1.55471900	2.45291000	-0.15281500
H	-2.36645500	1.84055700	1.04117300
C	4.14882200	-2.71332500	-3.04836000
C	3.21419900	-3.73095400	-3.07951600
H	3.41329700	-4.63794700	-3.64400000
H	5.08462000	-2.81593300	-3.59155400

Pu(IV)-L6

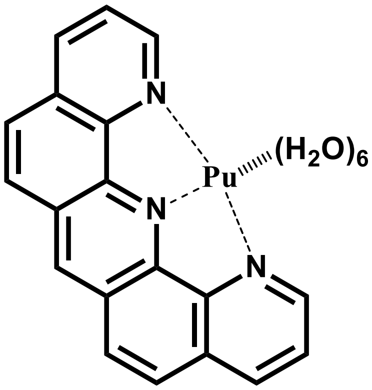

Pu	0.11079400	-0.08029000	0.25522900
O	-1.18470000	-0.01017600	-1.94133400
H	-2.09935400	0.32813300	-1.99032100
O	-0.68412700	-0.03628600	2.66782100
H	-1.27305100	-0.65061600	3.14464600
O	1.60968700	-1.28552600	1.80138400
H	1.35908100	-1.53088700	2.71186900
O	1.40154500	1.67829100	1.57953300
H	2.15732800	1.46838500	2.16118800
O	0.38976300	2.02573400	-1.22159800
H	1.03988900	2.75262100	-1.21507400
H	-0.47708500	0.68165500	3.29568700
H	1.22442100	2.63023400	1.70824800
H	2.42749800	-1.77363300	1.59174100
H	0.13126400	1.90662000	-2.15532500
H	-1.10996800	-0.66530400	-2.65963300
N	0.52726800	-2.22129100	-0.87653400
N	2.41548000	-0.27010900	-0.84012400
N	-1.78613700	-1.75187100	0.48829600
C	3.36197200	0.68647100	-0.82753700
C	4.58006800	0.59469700	-1.52138100
C	4.84872300	-0.54554500	-2.25593600
C	3.88846800	-1.59280900	-2.28562100
C	2.67373200	-1.40251800	-1.56424500
C	1.68311000	-2.43998600	-1.56154000

C	-2.95524900	-1.51953200	1.11121400
C	-3.98429500	-2.46825100	1.23158800
C	-3.79961200	-3.72589100	0.68743400
C	-2.59000000	-4.01478700	0.00118800
C	-1.60585300	-2.98585100	-0.08025800
C	-0.38927700	-3.22757800	-0.79954000
H	5.29414500	1.41045200	-1.46378600
H	5.78531300	-0.65181900	-2.79793600
H	-4.90129700	-2.20485200	1.74971800
H	-4.57117200	-4.48743600	0.77099900
H	-3.09115000	-0.53221800	1.54230100
H	3.15077100	1.56692600	-0.22988500
C	1.95616100	-3.66763900	-2.24128300
C	-0.19052700	-4.49721800	-1.42638000
C	0.99578900	-4.69171800	-2.15597700
H	1.17447000	-5.64307700	-2.65314600
O	-1.66425300	1.68152800	0.44757500
H	-1.55986500	2.47519400	-0.11259500
H	-2.39171800	1.86783900	1.07089100
C	4.11636300	-2.81585000	-2.98769100
C	3.18316600	-3.82820200	-2.95950100
H	3.37424200	-4.76334100	-3.47877400
H	5.04629600	-2.94558100	-3.53456600
C	-2.35465800	-5.27950900	-0.62152100
C	-1.19135900	-5.51491500	-1.31594600
H	-1.02459400	-6.47488500	-1.79679500
H	-3.11555700	-6.05189500	-0.55095400

PuO_2(VI)-L1

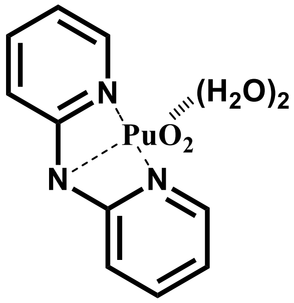

O	1.03365100	2.42175100	0.02090200
H	1.69876700	2.50617800	0.72767400
H	0.92706900	3.30408600	-0.37635300
N	0.01639200	-2.03724000	-1.18946000
N	-2.32775500	-1.13114900	-0.23970200
N	1.68504200	-0.05787200	-1.89523600
C	-3.57588000	-0.61843000	-0.19674200
C	-4.71048600	-1.41494600	-0.10272700
C	-4.55296600	-2.80105000	-0.06496400
C	-3.26696500	-3.33852200	-0.13982800
C	-2.17663500	-2.47767600	-0.22788900
C	-0.75631900	-2.96804000	-0.32528700
C	2.25522100	0.94164800	-2.60137600
C	3.35829200	0.74481000	-3.42300700
C	3.88933800	-0.54112600	-3.53277000
C	3.28540000	-1.58477300	-2.82991200
C	2.18543500	-1.31064800	-2.02197400

C	1.46327000	-2.37537200	-1.23980000
H	-5.69218500	-0.95510600	-0.07012500
H	-5.41723000	-3.45452800	0.00322500
H	-3.11147500	-4.41258000	-0.13880800
H	-0.71790200	-3.99340500	-0.71233800
H	-0.28327500	-2.97308600	0.66449200
H	3.78120400	1.58100300	-3.96907100
H	4.74776100	-0.73295600	-4.16910700
H	3.65699100	-2.60098200	-2.91390600
H	1.62406400	-3.36684700	-1.68007700
H	1.83299500	-2.41144800	-0.20761600
H	1.80694500	1.92368800	-2.50248600
H	-3.65909700	0.46134100	-0.24297100
H	-0.35994200	-2.09303500	-2.13987800
O	-1.72237600	1.65100000	1.19220300
H	-1.67815600	1.54958200	2.16006000
H	-2.29589900	2.41683700	1.01272900
Pu	-0.28739100	0.35701700	-0.36699400
O	0.47221000	-0.29137400	1.03363100
O	-1.04875400	1.05041700	-1.73958600

PuO_2(VI)-L2

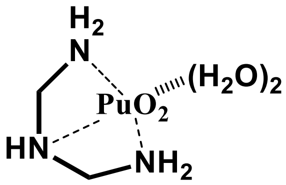

O	1.06342700	2.43120400	-0.07021900
H	1.59949600	2.74459800	0.68125400
H	0.94657500	3.19282000	-0.66746000
O	-1.79900000	1.73358800	1.01671500
H	-1.80002600	1.92384200	1.97284300
H	-2.41521900	2.36679900	0.60462300
Pu	-0.19071700	0.29939400	-0.20131000
O	0.50645300	-0.23397000	1.27518300
O	-0.88476400	0.80456100	-1.68832400
N	0.01155800	-2.04364500	-1.18965200
N	-2.20581500	-1.18235600	0.35081800
N	2.07898900	-0.10893000	-1.31805100
C	-2.24570300	-2.50030300	-0.35740900
C	-0.83165100	-3.04456500	-0.47305600
C	2.18851200	-1.38343400	-2.09491900
C	1.42917700	-2.47647300	-1.36194600
H	-0.83194500	-4.00297400	-1.00700400
H	-0.39014000	-3.21414300	0.51478900
H	1.48026600	-3.42019100	-1.91901500
H	1.85191300	-2.65362600	-0.36722000
H	-0.38503100	-1.93619400	-2.12917200
H	1.75472600	-1.20817300	-3.08489600
H	3.23527600	-1.67006400	-2.23949100
H	2.77109800	-0.12140500	-0.56384800
H	2.35055200	0.67407000	-1.91685300
H	-2.16391400	-1.35088600	1.35974200
H	-3.08970300	-0.69142900	0.19841000

H	-2.89171700	-3.21165000	0.16739400
H	-2.67776700	-2.33219300	-1.34933400

PuO_2(VI)-L3

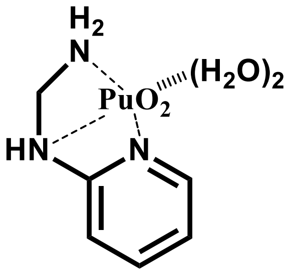

N	-3.51061100	-8.60971900	3.24079000
N	-5.68491100	-7.86510000	4.63233200
N	-1.58500700	-6.56734600	2.98317400
C	-6.96107100	-7.53204600	4.92553400
C	-7.87788900	-8.44642500	5.43019400
C	-7.46536000	-9.76250000	5.63931900
C	-6.15579900	-10.11956900	5.31397500
C	-5.29157900	-9.15058300	4.81181400
C	-3.87625300	-9.47304400	4.39961600
C	-1.11825500	-7.96660300	3.21300100
C	-2.16716000	-8.91072200	2.65089100
H	-8.89175800	-8.12878300	5.64823800
H	-8.15437200	-10.50362400	6.03253900
H	-5.81097600	-11.14119400	5.43783300
H	-3.78648700	-10.53312100	4.13360300
H	-3.17304900	-9.27944200	5.21826900
H	-2.24724800	-8.78002700	1.56661000
H	-1.88887800	-9.95471400	2.83323300
H	-7.24507000	-6.50105300	4.75141300
H	-4.21225000	-8.77578800	2.51585100
H	-0.14732000	-8.14828600	2.73886600
H	-0.99408200	-8.10269200	4.29155100
H	-0.92309000	-5.91470300	3.40871900
H	-1.55912600	-6.36129400	1.98044900
O	-2.81004700	-3.96089900	3.79113300
H	-2.36488700	-3.48073300	4.51252700
H	-2.93205900	-3.32449600	3.06340600
O	-5.54409500	-4.65849500	5.02083500
H	-5.58690000	-4.46403700	5.97491000
H	-6.13566300	-4.02576400	4.57444800
Pu	-3.91196600	-6.18562200	3.95072000
O	-3.18213400	-6.45932300	5.48080400
O	-4.61261200	-5.92019200	2.40630800

PuO_2(VI)-L4

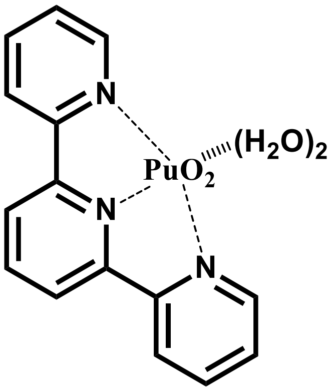

O	0.91546600	2.39810400	0.33228200
H	1.51422100	2.44720300	1.09895900
H	0.76762800	3.31090600	0.02771400
O	-1.78025100	1.55478500	1.33260100
H	-1.74162600	1.38996600	2.29181100
H	-2.30283500	2.36601400	1.20440600
Pu	-0.33831800	0.33255800	-0.30000200
O	0.36739400	-0.43223300	1.06336800
O	-1.04714300	1.13341400	-1.63929700
N	-0.01793300	-1.94112700	-1.29984700
N	1.75787200	0.06072600	-1.67703400
N	-2.38352800	-1.14191000	-0.25954900
C	2.51511500	1.11501200	-2.03560900
C	3.66439900	0.99881900	-2.81082900
C	4.04784700	-0.26908000	-3.24125500
C	3.26303000	-1.36500800	-2.88801500
C	2.12008100	-1.17654800	-2.10581700
C	1.21238800	-2.28800500	-1.75123000
C	-3.60035800	-0.65661900	0.05177200
C	-4.75654500	-1.42993300	0.03011200
C	-4.65087500	-2.76958600	-0.33652800
C	-3.39795700	-3.27817500	-0.67318400
C	-2.27671300	-2.44474100	-0.62865200
C	-0.93180100	-2.90614800	-1.03233500
H	4.23312400	1.88494300	-3.07051300
H	4.93414200	-0.40561100	-3.85281600
H	3.53025800	-2.35186900	-3.24492700
H	-5.71059500	-0.98342500	0.28800000
H	-5.52819800	-3.40759700	-0.37443600
H	-3.30624300	-4.30844800	-0.99414000
H	-3.64474100	0.38753300	0.33374900
H	2.18985900	2.08465200	-1.68135600
C	1.58134600	-3.62967400	-1.89313900
C	-0.61364800	-4.26226900	-1.15778800
C	0.66157300	-4.62467900	-1.57879600
H	2.57690100	-3.89829200	-2.22310600
H	0.93527000	-5.67158000	-1.66262200
H	-1.33985200	-5.02623500	-0.91023100

PuO_2(VI)-L5

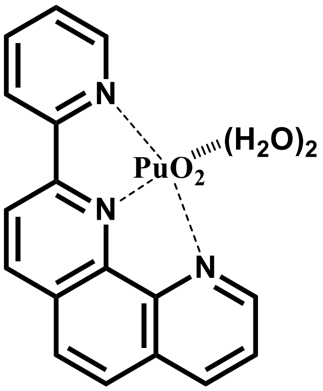

O	0.61564900	2.45311000	-0.21144900
H	1.06577100	2.85345100	0.55363500
H	0.38224200	3.17824400	-0.81813600
O	-1.88726900	1.52278100	1.05036700
H	-1.84787000	1.53501700	2.02352000
H	-2.45068500	2.26850600	0.77841600
Pu	-0.32644300	0.13116900	-0.34745500
O	0.38200800	-0.32343900	1.14608100
O	-1.03810100	0.61192400	-1.83065700
N	0.12033900	-2.19612400	-1.08815200
N	1.91616600	-0.15752600	-1.49066700
N	-2.27081200	-1.46897400	-0.06322200
C	2.77152100	0.83529700	-1.75046100
C	4.04784000	0.63178000	-2.29745000
C	4.45595300	-0.65610200	-2.57738500
C	3.57692300	-1.73394600	-2.32648200
C	2.29797200	-1.43596800	-1.78756900
C	1.36297200	-2.50283200	-1.55972300
C	-3.48299500	-1.05033400	0.34473300
C	-4.59230500	-1.88671100	0.43030900
C	-4.44249600	-3.22274300	0.07048500
C	-3.19337100	-3.66609600	-0.35926700
C	-2.11872600	-2.77312500	-0.41799500
C	-0.77902600	-3.17732900	-0.89129700
H	4.69069800	1.48468600	-2.48529400
H	5.44078300	-0.85133600	-2.99195400
H	-5.54367500	-1.48972700	0.76705800
H	-5.28094200	-3.91056600	0.11525200
H	-3.07181600	-4.69858900	-0.66019400
H	-3.56185400	-0.00634200	0.61796500
H	2.43714400	1.83562200	-1.50787400
C	1.75152000	-3.83737000	-1.85017700
C	-0.44938200	-4.52785400	-1.13723100
C	0.80248900	-4.85338800	-1.60960700
H	1.06358500	-5.89006600	-1.80135800
H	-1.16965200	-5.31279600	-0.94855500
C	3.93570900	-3.09409700	-2.60499200
C	3.05765100	-4.10895700	-2.36955000
H	3.32957700	-5.13847500	-2.58080400
H	4.92174100	-3.29810100	-3.01088700

PuO_2(VI)-L6

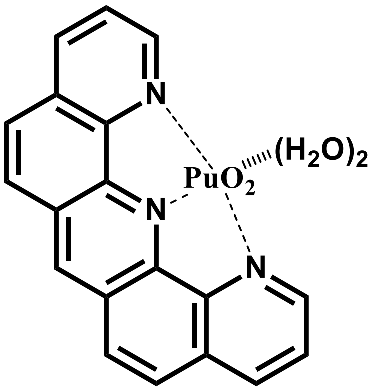

O	0.95258800	2.54223000	-0.86372800
H	1.34647400	3.04644400	-0.12961800
H	0.89596000	3.14453900	-1.62677400
O	-1.87519100	2.15280700	-0.07914300
H	-1.93982200	2.61884100	0.77341200
H	-2.38592700	2.66972500	-0.72740300
Pu	-0.23161100	0.34239200	-0.63903600
O	0.25389600	0.27842200	1.00318500
O	-0.72042200	0.43781900	-2.27873800
N	0.04821900	-2.10160500	-0.84106900
N	2.10386800	-0.37807300	-1.37324500
N	-2.33196500	-0.99356100	-0.07505800
C	3.11467000	0.45516500	-1.63731000
C	4.38630200	0.02102300	-2.04118400
C	4.61949200	-1.33290800	-2.17664100
C	3.57713600	-2.24661200	-1.90721700
C	2.32378800	-1.72014100	-1.50484600
C	1.23724100	-2.62055200	-1.22254000
C	-3.49922100	-0.46285300	0.30121900
C	-4.63319900	-1.23116000	0.60528600
C	-4.55357200	-2.60645000	0.51546100
C	-3.33757000	-3.20653400	0.12153500
C	-2.24219800	-2.35385200	-0.16667300
C	-0.98648600	-2.92918500	-0.57068700
H	5.16208400	0.75243700	-2.23942900
H	5.59244400	-1.70267900	-2.48718700
H	-5.54980600	-0.73479100	0.90453900
H	-5.41268800	-3.23067500	0.74363900
H	-3.54036500	0.61671800	0.36515100
H	2.91103100	1.51209200	-1.52459100
C	1.44364400	-4.02798800	-1.35248100
C	-0.86884200	-4.34898800	-0.67406000
C	0.36582500	-4.87272400	-1.06970400
H	0.48916300	-5.94894300	-1.15849900
C	3.75178300	-3.66882200	-2.02841400
C	2.72875700	-4.52368000	-1.76226400
H	2.86389800	-5.59685000	-1.85459500
H	4.72164100	-4.04565100	-2.33802200
C	-3.18582000	-4.63198000	0.00776600
C	-2.00187800	-5.18042800	-0.37391400
H	-1.89061300	-6.25693700	-0.45892100
H	-4.04031800	-5.26213000	0.23409600

Structural chemical formulas and its coordinates of optimized structures (TPSSH/def2-TZVP)

Pu(IV)-L1

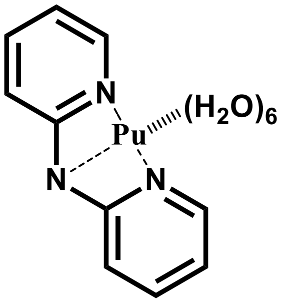

Pu	0.03704900	-0.61307900	0.07094500
O	0.17121200	0.18803100	-2.36880000
H	0.98933500	0.38128800	-2.85864100
O	1.55385400	-1.18143200	2.03947400
H	2.44226600	-0.82363400	2.20835100
O	-0.89454500	0.06109300	2.26651000
H	-0.41300600	0.00828000	3.10939500
O	-1.07262300	-2.57371200	1.22111500
H	-1.75003200	-2.54234700	1.91827200
O	-0.98320700	-2.16483000	-1.64076500
H	-1.53606700	-2.95072000	-1.49307800
H	1.41519100	-1.86794200	2.71523500
H	-0.85843000	-3.51600400	1.11031300
H	-1.79109000	0.36839100	2.47868000
H	-0.91931900	-2.06341700	-2.60522100
H	-0.55396000	0.41173300	-2.97770100
N	0.01849600	1.88907700	0.07001800
N	2.23134000	0.49103700	-0.29580800
N	-2.22873900	0.44257900	-0.21562700
C	3.27847500	-0.14061100	-0.89248100
C	4.53448600	0.43034300	-1.00176100
C	4.74393600	1.70606000	-0.47736200
C	3.67324800	2.37232400	0.11683900
C	2.43383300	1.75115100	0.18175400
C	1.22425600	2.43474300	0.75215600
C	-3.31610500	-0.29842700	-0.54132200
C	-4.59333300	0.23598000	-0.67399900
C	-4.77713400	1.60406300	-0.47691200
C	-3.67271000	2.37771600	-0.13710800
C	-2.42209800	1.77512600	0.00458500
C	-1.23605300	2.56795900	0.46474100
H	5.33342000	-0.11997600	-1.48553100
H	5.71691800	2.18163900	-0.54766200
H	3.79524800	3.37717500	0.50651100
H	1.28613200	3.51865200	0.60994900
H	1.12467200	2.26176000	1.82883400
H	-5.41874000	-0.41368600	-0.94257800
H	-5.75786300	2.05585300	-0.58513100
H	-3.77560700	3.44420500	0.03360700
H	-1.26387800	3.58105200	0.04649600
H	-1.24651400	2.68753100	1.55278000
H	-3.15585800	-1.35397700	-0.71091600

H	3.09277500	-1.13298100	-1.28312100
H	0.14364300	2.03604400	-0.93435400
O	1.39649300	-2.69181000	-0.36455400
H	2.12513700	-3.04695800	0.17260500
H	1.30363200	-3.29253300	-1.12391400

Pu(IV)-L2

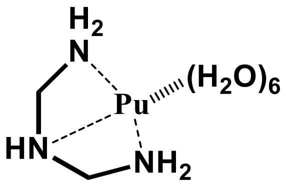

Pu	0.40388700	-0.00294700	0.02033100
O	0.11008700	-1.10064700	2.27309300
H	0.87614600	-1.53260900	2.69406700
O	1.85663200	-1.39384600	-1.56418400
H	1.88965500	-2.34402200	-1.78123900
O	-0.37882100	0.02467500	-2.31968600
H	0.13027600	-0.43375100	-3.01305000
O	2.12457800	1.50625200	-0.99048300
H	2.05684700	2.12114100	-1.74492600
O	0.89228700	1.53203300	1.92992800
H	1.29893900	2.41837600	1.95118000
H	2.61167200	-1.00635300	-2.04405300
H	3.04578100	1.59325100	-0.68276200
H	-1.16131200	0.38283100	-2.77586000
H	0.70001600	1.32000200	2.86168600
H	-0.64493700	-1.33691900	2.84174700
N	-2.17975500	-0.00502500	0.31462600
N	-0.65894000	-2.28909400	-0.29994400
N	-0.68244100	2.25136300	-0.43076600
C	-2.06524600	-2.45968600	0.21185400
C	-2.86702100	-1.24294000	-0.18168600
C	-2.02557600	2.44216000	0.22151900
C	-2.88162600	1.24859800	-0.12298500
H	-3.88027500	-1.29329500	0.23426300
H	-2.96649600	-1.16051500	-1.26606400
H	-3.85936200	1.31254700	0.36854000
H	-3.06949300	1.17638700	-1.19724000
H	-2.23663900	-0.01871100	1.33960600
H	-1.87250800	2.53579300	1.29882800
H	-2.48735000	3.37120800	-0.12652900
H	-0.79337400	2.41357300	-1.43520700
H	-0.07097100	3.01374300	-0.12774400
H	-0.67939200	-2.48149700	-1.30503500
H	-0.08687100	-3.03702600	0.10483400
H	-2.50885800	-3.36784500	-0.20747100
H	-2.02468600	-2.59513200	1.29352700
O	2.62971300	-0.58534000	1.01123200
H	3.25500800	-1.25752800	0.68286700
H	3.08216400	-0.15357600	1.75947400

Pu(IV)-L3

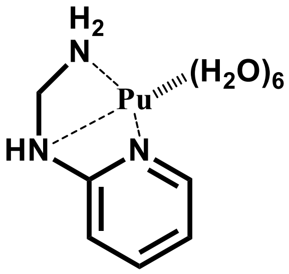

Pu	-0.65851800	-0.32025900	-0.02745000
O	-0.35005300	0.65778900	2.23677400
H	-0.65527600	1.46656400	2.68095600
O	-2.74306300	-0.89524500	-1.39403500
H	-3.60097100	-0.44956300	-1.50831800
O	-0.24487100	-0.31543500	-2.51921400
H	-0.95653700	-0.28006300	-3.18268100
O	-0.45387800	-2.67131200	-0.89891000
H	-0.33497400	-2.95405500	-1.82218200
O	0.20360100	-1.95648100	1.77705200
H	1.10724300	-2.25503200	1.97540300
H	-2.86322600	-1.76660000	-1.81060100
H	-0.45946300	-3.49098500	-0.37456600
H	0.58219000	-0.37026000	-3.02929400
H	-0.37820700	-2.51758100	2.31871500
H	0.08839700	0.12631800	2.92336300
O	-2.51431900	-1.17709600	1.43516600
H	-3.29564800	-1.66556100	1.12254200
H	-2.67812100	-1.00729200	2.37907400
N	0.42951300	1.94508600	-0.63179800
N	1.92191500	-0.21988100	-0.12294000
N	-2.22922100	1.68194700	0.16987000
C	2.70875900	-1.30103800	-0.28754100
C	4.11009700	-1.25450600	-0.20720300
C	4.73430600	-0.04139500	0.09816900
C	3.93774600	1.08166900	0.26823200
C	2.54287500	0.97519700	0.11231200
C	1.67616200	2.19104500	0.13053700
C	-1.57314100	3.02325600	0.35083700
C	-0.48502400	3.14604800	-0.68903700
H	4.68221900	-2.16664900	-0.34257200
H	5.81494600	0.02381600	0.18016700
H	4.38174100	2.05337400	0.45940200
H	2.21781600	3.04973000	-0.28729600
H	1.43491000	2.46629900	1.16535800
H	-0.91653700	3.17922700	-1.69219000
H	0.08712200	4.06730400	-0.55356900
H	2.21272100	-2.24969000	-0.46101900
H	0.71975300	1.76506800	-1.59377900
H	-2.30384800	3.82899600	0.24326000
H	-1.17697700	3.08929700	1.36531800
H	-2.92405500	1.54966500	0.90920100
H	-2.79268400	1.75298800	-0.68284700

Pu(IV)-L4

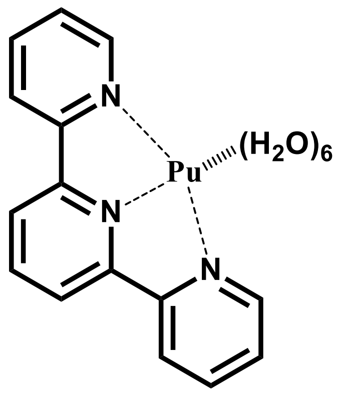

Pu	0.00601600	-0.93839200	0.08555600
O	0.14760200	-0.38252900	-2.43555500
H	0.96920800	-0.19325100	-2.91822700
O	1.53258200	-1.40166300	2.10395300
H	2.41210700	-1.04911900	2.31724800
O	-0.97140600	-0.20303900	2.30730800
H	-0.43158100	-0.05788200	3.10191100
O	-1.04405400	-2.85802800	1.42561400
H	-1.59139200	-2.71872700	2.21575700
O	-1.12986500	-2.61325300	-1.46004700
H	-1.67379800	-3.39684000	-1.27549000
H	1.34946900	-2.07527300	2.78049400
H	-0.93005400	-3.81975000	1.34789500
H	-1.83308800	0.20311900	2.49192600
H	-1.08817300	-2.53548900	-2.42696400
H	-0.56059400	-0.03859700	-3.00515600
N	0.01607800	1.61502900	0.00224000
N	-2.25888400	0.19698800	-0.24801600
N	2.27567700	0.19051800	-0.32732900
C	-3.38020300	-0.52143800	-0.40658600
C	-4.65869300	0.04059600	-0.53344800
C	-4.78367400	1.42227100	-0.49705800
C	-3.63172100	2.18464200	-0.31928000
C	-2.38002100	1.55901600	-0.18940000
C	-1.14397600	2.31431600	-0.00007200
C	3.37731800	-0.49750000	-0.66288300
C	4.65738900	0.06805100	-0.74929200
C	4.81496300	1.41467400	-0.44693400
C	3.68036400	2.15217500	-0.12547500
C	2.41719100	1.53544600	-0.09730600
C	1.18492300	2.29744800	0.07815200
H	-5.51801800	-0.60701700	-0.66774400
H	-5.75017800	1.90371100	-0.60237100
H	-3.72118600	3.26198700	-0.28845600
H	5.49838800	-0.55414500	-1.03515000
H	5.79031500	1.88871100	-0.47720500
H	3.78722200	3.20912000	0.07595400
H	3.25354900	-1.55012000	-0.88271300
H	-3.26906500	-1.59653300	-0.44953700
C	-1.16418900	3.72328100	0.15249300
C	1.21229200	3.70391800	0.25229200

C	0.02534000	4.41783800	0.30313000
H	-2.09964300	4.26557000	0.15877100
H	0.02798500	5.49403300	0.43910700
H	2.15157400	4.23140800	0.34419600
O	1.41133600	-3.04826400	-0.29362900
H	1.27215800	-3.66415500	-1.03294000
H	2.06721000	-3.47275600	0.28417000

Pu(IV)-L5

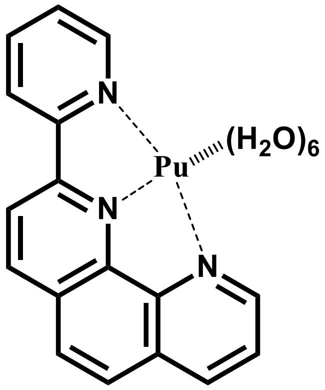

Pu	-0.53368100	-0.95281200	-0.00404600
O	-0.84110000	-0.15895400	2.42562000
H	-1.64385000	-0.33855600	2.94191800
O	-2.38619600	-1.53413200	-1.73120800
H	-3.06140300	-0.98386200	-2.15999000
O	0.19682100	-0.76055300	-2.38734300
H	-0.42303300	-0.84468200	-3.12955700
O	-0.15482200	-3.33500800	-0.96045900
H	0.25422600	-3.54482500	-1.81578500
O	0.37562800	-2.52085300	1.84901700
H	0.82883700	-3.37842700	1.84134400
H	-2.53075400	-2.43417300	-2.06535600
H	-0.47072600	-4.18400000	-0.61037800
H	1.04876500	-0.50385600	-2.77259800
H	0.49955200	-2.16177300	2.74225400
H	-0.42274200	0.60004000	2.86182300
N	0.43413200	1.43056100	-0.03254100
N	2.04735400	-0.75955000	-0.04770300
N	-2.21442000	0.96142900	-0.01967500
C	2.85851000	-1.83885900	-0.07571600
C	4.24861200	-1.76948300	-0.05658100
C	4.85270300	-0.52247700	-0.01364700
C	4.03642400	0.62837300	-0.00390000
C	2.62919200	0.45892700	-0.02050200
C	1.76601700	1.62432400	-0.03030800
C	-3.52741200	0.71084900	0.07867500
C	-4.51281300	1.70104300	0.11399200
C	-4.11996200	3.03020000	0.03535700
C	-2.76041000	3.31189000	-0.04861500
C	-1.82208700	2.26995600	-0.06228000
C	-0.38340500	2.52447000	-0.08420700
H	4.83630900	-2.67964900	-0.07765600
H	5.93394600	-0.42687200	0.00260900
H	-5.55676200	1.42071600	0.19524600

H	-4.84917500	3.83268800	0.04820600
H	-2.44435400	4.34466400	-0.09131100
H	-3.82046200	-0.33064800	0.13159300
H	2.36862700	-2.80363100	-0.12174500
C	2.35998200	2.92578800	-0.05163800
C	0.13566900	3.83631100	-0.13398600
C	1.49054400	4.04229500	-0.10872600
H	1.89844700	5.04756500	-0.13636900
H	-0.52595500	4.68850500	-0.18801600
O	-2.31162400	-2.43996500	1.10017400
H	-2.09833900	-2.98491300	1.87487100
H	-3.20744700	-2.70222900	0.83409600
C	4.58671800	1.94177400	0.00778100
C	3.76079900	3.06448900	-0.02368200
H	4.20102000	4.05649900	-0.03057900
H	5.66515200	2.06625200	0.03127300

Pu(IV)-L6

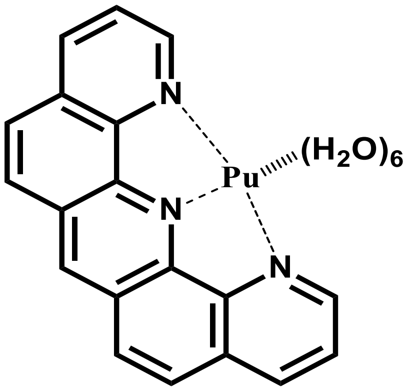

Pu	0.02948200	-1.26078800	-0.00100200
O	-0.54282300	-0.68621200	2.45055800
H	-1.19876200	-1.17836000	2.96993000
O	-1.48670300	-2.46517200	-1.73794600
H	-2.31240800	-2.20966200	-2.17859700
O	0.65504400	-0.78147200	-2.38482500
H	0.12249200	-1.07551700	-3.14054300
O	1.24698000	-3.33487100	-1.00881500
H	1.72268200	-3.35192500	-1.85469300
O	1.50086300	-2.41473700	1.80322700
H	2.22918500	-3.05347300	1.75896000
H	-1.26594200	-3.34495700	-2.08322300
H	1.27150100	-4.24814500	-0.68070200
H	1.36057900	-0.22563700	-2.74903600
H	1.51287100	-2.06111600	2.70673500
H	-0.42284400	0.15407000	2.91963100
N	-0.01364000	1.32047400	-0.00622200
N	2.33919800	-0.05151400	-0.03843600
N	-2.32568100	-0.13384000	0.01086200
C	3.50898700	-0.70558900	-0.06800800
C	4.76447900	-0.08208500	-0.05680700
C	4.82215500	1.29193300	-0.01271000
C	3.61159800	2.02817500	0.00535700
C	2.38190300	1.30584000	-0.00747400
C	1.13795600	2.02670700	-0.00567900

C	-3.47480600	-0.81937900	0.08325900
C	-4.74870800	-0.23445400	0.10720000
C	-4.84860400	1.13593600	0.04400300
C	-3.66134100	1.90625100	-0.01440400
C	-2.40910700	1.22231900	-0.01765800
C	-1.19000500	1.98454400	-0.03086400
H	5.66353400	-0.68609600	-0.08292000
H	5.77388200	1.81313700	0.00051100
H	-5.62759900	-0.86509400	0.16829000
H	-5.81526800	1.62894800	0.04620600
H	-3.38894200	-1.89902100	0.12451100
H	3.45312300	-1.78709500	-0.10874900
C	1.16409300	3.45382200	-0.01326000
C	-1.26381600	3.41056700	-0.05534800
C	-0.06239500	4.13417000	-0.04307300
H	-0.08182200	5.22017200	-0.05563400
O	-1.07294700	-3.32821700	1.09043200
H	-0.66203900	-3.77987400	1.84476000
H	-1.81050800	-3.89595600	0.81688900
C	3.59905000	3.44636900	0.01952300
C	2.40449000	4.14557900	0.00529100
H	2.40959400	5.23055000	0.00579500
H	4.54134300	3.98441400	0.03212700
C	-3.69610000	3.32374600	-0.05462500
C	-2.52624600	4.06143600	-0.07711900
H	-2.56655400	5.14533800	-0.10259200
H	-4.65607700	3.82962400	-0.05912000

PuO_2(VI)-L1

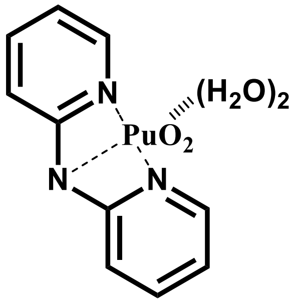

O	-1.53633300	-2.62591300	0.52332400
H	-1.87910400	-2.84682300	1.40260400
H	-1.81288500	-3.33718700	-0.07324000
N	-0.00166600	1.80940400	0.18273100
N	2.20401500	0.40791300	-0.28027000
N	-2.20457100	0.40381100	-0.28129900
C	3.19858700	-0.21036100	-0.94361000
C	4.44476900	0.36606000	-1.12168300
C	4.67191200	1.63792100	-0.60691200
C	3.63689000	2.29527000	0.04922300
C	2.41478100	1.65439300	0.19295500
C	1.22990300	2.28663800	0.85902800
C	-3.19762100	-0.21641100	-0.94509400
C	-4.44472600	0.35773400	-1.12406100
C	-4.67442300	1.62932600	-0.60976700
C	-3.64098900	2.28867600	0.04687800
C	-2.41784000	1.65001400	0.19154600

C	-1.23450500	2.28440600	0.85831200
H	5.21389200	-0.17158500	-1.66086200
H	5.63393400	2.12011000	-0.73259000
H	3.77067800	3.29828900	0.43536100
H	1.29696900	3.37836000	0.83405400
H	1.16989500	1.98020900	1.90786900
H	-5.21257500	-0.18141700	-1.66355000
H	-5.63721800	2.10978100	-0.73616300
H	-3.77682900	3.29153500	0.43271300
H	-1.30352100	3.37600500	0.83334300
H	-1.17456800	1.97803000	1.90717200
H	-2.97606600	-1.19660100	-1.34727000
H	2.97906800	-1.19083500	-1.34622400
H	-0.00169600	2.15073600	-0.77998100
O	1.54234400	-2.62170100	0.52492600
H	1.88612100	-2.84081600	1.40425800
H	1.82077100	-3.33265600	-0.07114200
Pu	0.00062800	-0.73361400	0.10744500
O	-0.00003900	-0.46883400	1.80690100
O	0.00132300	-1.02107600	-1.58355900

PuO_2(VI)-L2

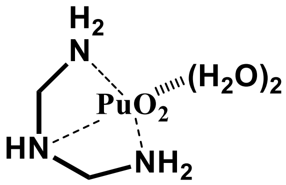

O	2.39192800	1.56807800	-0.08102600
H	2.85421000	1.87011700	-0.87863500
H	2.92406700	1.85894400	0.67623700
O	2.38902400	-1.57007200	-0.07665800
H	2.85229700	-1.87484800	-0.87264800
H	2.91923900	-1.85993200	0.68233900
Pu	0.48841500	0.00085700	0.00656000
O	0.39100300	-0.00000800	-1.70640700
O	0.56576200	0.00174800	1.71971800
N	-2.05399900	0.00354000	0.20662900
N	-0.52847800	-2.33825100	-0.02318400
N	-0.52385000	2.34200100	-0.02554700
C	-1.98383700	-2.42733600	0.29922100
C	-2.68381700	-1.23823300	-0.32028300
C	-1.97904900	2.43434000	0.29665200
C	-2.68134100	1.24598100	-0.32165300
H	-3.75352400	-1.25338700	-0.08885100
H	-2.57554000	-1.23557400	-1.40747700
H	-3.75103400	1.26347900	-0.09032500
H	-2.57298600	1.24196100	-1.40883600
H	-2.18431800	0.00423600	1.22142900
H	-2.07997100	2.42078400	1.38396500
H	-2.40438800	3.37238600	-0.06647000
H	-0.37148500	2.69109600	-0.97275400
H	-0.00242800	2.97124200	0.58438500
H	-0.37685200	-2.68877700	-0.96998100
H	-0.00823600	-2.96776600	0.58747100

H	-2.41107500	-3.36490900	-0.06289300
H	-2.08465000	-2.41244200	1.38652500

PuO_2(VI)-L3

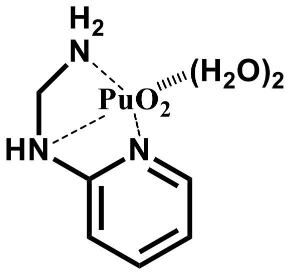

N	-0.29927000	1.90461700	0.50989200
N	-1.84383800	-0.19950500	0.14257800
N	2.39856700	1.43430400	0.01650600
C	-2.64828500	-1.24336000	0.41902200
C	-4.02589500	-1.17063600	0.29778100
C	-4.59808000	0.02561600	-0.12082800
C	-3.77316300	1.11517000	-0.37782100
C	-2.40062000	0.97182100	-0.23216300
C	-1.43751000	2.10793900	-0.42489900
C	1.82945500	2.75115500	-0.38408400
C	0.67089000	3.04071600	0.54348900
H	-4.63177000	-2.03654100	0.53143900
H	-5.67261700	0.11620300	-0.22589400
H	-4.18905900	2.07104400	-0.67197100
H	-1.92800400	3.06694000	-0.23240800
H	-1.05469600	2.12908000	-1.44957900
H	1.02656600	3.13749000	1.57170400
H	0.17450200	3.97707200	0.27592900
H	-2.16718000	-2.15642400	0.74415900
H	-0.69405200	1.79953800	1.44455700
H	2.57873300	3.54476200	-0.32780800
H	1.50048100	2.66933800	-1.42143200
H	3.14805800	1.17612800	-0.62467700
H	2.83939200	1.51931100	0.93350900
O	2.88906700	-1.48098900	-0.24590900
H	3.29830100	-1.78046700	-1.07213800
H	3.41627700	-1.85228100	0.47815500
O	0.11889400	-2.78979000	-0.38889800
H	-0.08929900	-3.16717000	-1.25757300
H	0.22495000	-3.53339700	0.22400700
Pu	0.65900100	-0.41371800	0.01862500
O	0.54146300	-0.13684400	-1.67126900
O	0.79980000	-0.67168000	1.70831200

PuO_2(VI)-L4

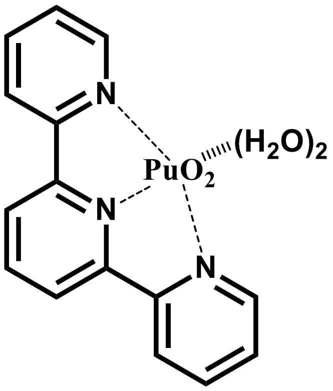

O	-1.50901300	-2.87756800	0.75121500
H	-1.82710400	-3.01120300	1.65683600
H	-1.73744600	-3.67518000	0.25150600
O	1.51416500	-2.87128200	0.75892400
H	1.83034600	-2.99968800	1.66597100
H	1.74704200	-3.67019600	0.26337800
Pu	0.00038400	-0.99727100	0.13331000
O	-0.00124900	-0.60253100	1.80267200
O	0.00201300	-1.44334000	-1.52040900
N	-0.00142600	1.51434400	-0.00251300
N	-2.20532500	0.07524100	-0.32442400
N	2.20464100	0.07850100	-0.32284400
C	-3.24623600	-0.66334700	-0.73794800
C	-4.51059800	-0.13421300	-0.94697400
C	-4.70692600	1.22205300	-0.71968700
C	-3.62850500	1.99880600	-0.31830000
C	-2.38211100	1.40414500	-0.13785900
C	-1.16759700	2.16877900	0.17854900
C	3.24675100	-0.65830500	-0.73652000
C	4.51063100	-0.12734200	-0.94387200
C	4.70515400	1.22886500	-0.71468100
C	3.62538700	2.00386900	-0.31353200
C	2.37955600	1.40746500	-0.13492700
C	1.16360500	2.17032800	0.18030500
H	-5.31385600	-0.77584400	-1.28496100
H	-5.67938400	1.67436800	-0.87160500
H	-3.75378000	3.06331300	-0.17619900
H	5.31491100	-0.76756400	-1.28210200
H	5.67720200	1.68253100	-0.86518700
H	3.74918100	3.06839700	-0.17032300
H	3.05915900	-1.70926600	-0.90950500
H	-3.05708800	-1.71434800	-0.90882600
C	-1.19848200	3.49801600	0.60073800
C	1.19207200	3.49956500	0.60268600
C	-0.00382200	4.16294000	0.83038000
H	-2.14127400	4.00228400	0.76048100
H	-0.00477600	5.18933000	1.17607100
H	2.13394100	4.00504900	0.76400200

PuO_2(VI)-L5

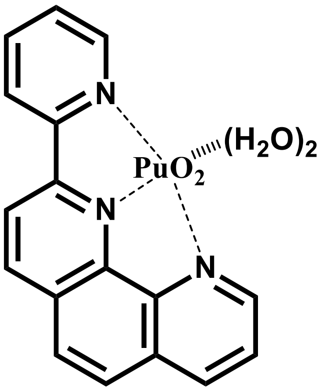

O	-0.01005700	-3.51813900	-0.04251400
H	-0.10657200	-4.11921700	0.71099800
H	0.01963700	-4.06742900	-0.84016300
O	2.55118800	-2.34052100	0.85155200
H	2.83296000	-2.28776000	1.77699300
H	3.01494500	-3.09506100	0.46079800
Pu	0.53236600	-1.05008100	0.01561100
O	0.28630600	-0.88834800	1.70835200
O	0.77184200	-1.25319200	-1.67477600
N	-0.29889000	1.29112300	-0.03329000
N	-1.95737100	-0.85219100	-0.23651100
N	2.29195500	0.71903200	-0.15414400
C	-2.77150100	-1.89534200	-0.39450800
C	-4.16552200	-1.78568000	-0.40987200
C	-4.73916100	-0.54616500	-0.24275500
C	-3.91228100	0.58237500	-0.07815700
C	-2.51267200	0.38164400	-0.09225000
C	-1.63759800	1.50605300	0.03459200
C	3.57288100	0.38228400	-0.37139800
C	4.59179400	1.31440800	-0.49330000
C	4.27116400	2.66151700	-0.38833100
C	2.94595500	3.02170900	-0.18546000
C	1.96968700	2.03279300	-0.08018600
C	0.53839400	2.33470400	0.05815900
H	-4.76752500	-2.67413500	-0.54892500
H	-5.81672400	-0.42744700	-0.23995000
H	5.60712800	0.98381300	-0.66897900
H	5.03658000	3.42285000	-0.47662900
H	2.67824400	4.06721600	-0.13030500
H	3.78676200	-0.67530900	-0.44607400
H	-2.30247500	-2.86306800	-0.51027200
C	-2.19092800	2.79783900	0.20152100
C	0.06053600	3.64420400	0.24851100
C	-1.29104600	3.87339000	0.32241100
H	-1.67031400	4.87834200	0.46806100
H	0.75193800	4.46934400	0.33972200
C	-4.43742500	1.90076500	0.09385900
C	-3.60725600	2.96780600	0.23480200
H	-4.00710100	3.96635700	0.36523200
H	-5.51294200	2.03197800	0.10731500

PuO_2(VI)-L6

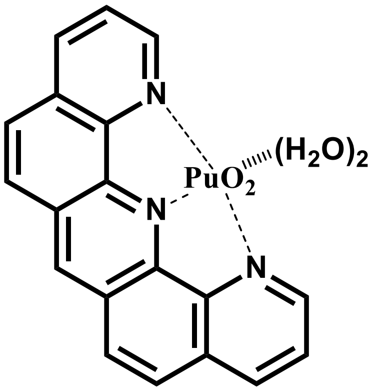

O	1.39698200	-3.41106900	-0.45457800
H	1.63481600	-3.69097600	-1.35118400
H	1.61010100	-4.14748200	0.13695700
O	-1.39982200	-3.40419200	0.47301200
H	-1.60897500	-4.14936300	-0.10885700
H	-1.63999200	-3.67224500	1.37260300
Pu	0.00017200	-1.34477200	-0.00061500
O	-0.13171000	-1.36002700	-1.71421500
O	0.13271800	-1.34650400	1.71295900
N	-0.00006900	1.12114700	-0.00143600
N	2.30080500	-0.29951400	-0.00694600
N	-2.30022300	-0.29990900	-0.00398900
C	3.43539500	-0.99799300	0.02704900
C	4.70034400	-0.40326600	0.06426100
C	4.79499400	0.97032900	0.06868500
C	3.61888200	1.74318700	0.04445700
C	2.38395900	1.05809900	0.01207100
C	1.16157400	1.80398900	0.00874400
C	-3.43434600	-0.99870300	-0.04639300
C	-4.69939000	-0.40423700	-0.08432300
C	-4.79466700	0.96935400	-0.08020500
C	-3.61900900	1.74257900	-0.04802200
C	-2.38392400	1.05776000	-0.01633000
C	-1.16180800	1.80391600	-0.00786000
H	5.58199300	-1.03047500	0.08718300
H	5.76119900	1.46119200	0.09412600
H	-5.58069500	-1.03163000	-0.11437600
H	-5.76103900	1.45989900	-0.10545000
H	-3.33994300	-2.07614500	-0.04704400
H	3.34143600	-2.07545600	0.02150000
C	1.21087800	3.22635700	0.01756000
C	-1.21146700	3.22627000	-0.00919900
C	-0.00036300	3.91828000	0.00618800
H	-0.00041500	5.00318600	0.00905300
C	3.63592400	3.17628300	0.05305800
C	2.48111300	3.88784000	0.03739800
H	2.49912200	4.97119600	0.04207700
H	4.59483600	3.68043200	0.07160600
C	-3.63647100	3.17571500	-0.04852200
C	-2.48190900	3.88749200	-0.02687200
H	-2.50022900	4.97085200	-0.02563500
H	-4.59549100	3.67969100	-0.06608700
